# Global Profiling of N-Glycoproteins and N-Glycans in the Diatom *Phaeodactylum tricornutum*

**DOI:** 10.3389/fpls.2021.779307

**Published:** 2021-12-03

**Authors:** Xihui Xie, Hong Du, Jichen Chen, Muhammad Aslam, Wanna Wang, Weizhou Chen, Ping Li, Hua Du, Xiaojuan Liu

**Affiliations:** ^1^Guangdong Provincial Key Laboratory of Marine Biotechnology, STU-UNIVPM Joint Algal Research Center, College of Sciences, Institute of Marine Sciences, Shantou University, Shantou, China; ^2^Southern Marine Science and Engineering Guangdong Laboratory, Guangzhou, China; ^3^Faculty of Marine Sciences, Lasbela University of Agriculture, Water & Marine Sciences, Uthal, Pakistan

**Keywords:** N-glycosylation modification, N-glycoprotein, N-glycan, *Phaeodactylum tricornutum*, biopharmaceuticals

## Abstract

N-glycosylation is an important posttranslational modification in all eukaryotes, but little is known about the N-glycoproteins and N-glycans in microalgae. Here, N-glycoproteomic and N-glycomic approaches were used to unveil the N-glycoproteins and N-glycans in the model diatom *Phaeodactylum tricornutum*. In total, 863 different N-glycopeptides corresponding to 639 N-glycoproteins were identified from *P. tricornutum*. These N-glycoproteins participated in a variety of important metabolic pathways in *P. tricornutum*. Twelve proteins participating in the N-glycosylation pathway were identified as N-glycoproteins, indicating that the N-glycosylation of these proteins might be important for the protein N-glycosylation pathway. Subsequently, 69 N-glycans corresponding to 59 N-glycoproteins were identified and classified into high mannose and hybrid type N-glycans. High mannose type N-glycans contained four different classes, such as Man-5, Man-7, Man-9, and Man-10 with a terminal glucose residue. Hybrid type N-glycan harbored Man-4 with a terminal GlcNAc residue. The identification of N-glycosylation on nascent proteins expanded our understanding of this modification at a N-glycoproteomic scale, the analysis of N-glycan structures updated the N-glycan database in microalgae. The results obtained from this study facilitate the elucidation of the precise function of these N-glycoproteins and are beneficial for future designing the microalga to produce the functional humanized biopharmaceutical N-glycoproteins for the clinical therapeutics.

## Introduction

Diatoms are a group of unicellular eukaryotic microalgae and contribute approximately 32% of global phytoplankton primary production (Uitz et al., 2010). *Phaeodactylum tricornutum*, a pennate diatom, is an ideal model organism for basic research of biotechnology, due to availability of its complete sequenced genome (Bowler et al., 2008) and convenient genetic operating systems (e.g., established overexpression, knock-down, and knock-out of target genes) (Daboussi et al., 2014; Weyman et al., 2015; Nymark et al., 2016). In addition, *P. tricornutum* has been used as an alternative model organism for the expression of recombinant biopharmaceutical N-glycoproteins (Hempel et al., 2017; Vanier et al., 2018). Especially, a recombinant monoclonal antibody directed against the Hepatitis B virus surface antigen was reported to be modified by high mannose type N-glycans (Vanier et al., 2018). However, the N-glycosylation pathway in *P. tricornutum*, a major post-translational modification in the maturation of proteins, has received very little attention. Mathieu-rivet et al. (2013) demonstrated that protein N-glycosylation in *Chlamydomonas reinhardtii* occurs in the endoplasmic reticulum (ER) and Golgi apparatus. The initial steps of N-glycosylation pathway in the ER are highly conserved among almost all the eukaryotes. An oligosaccharide intermediate (Man_5_GlcNAc_2_-PP-Dol) first synthesizes on the cytosolic side of the ER, and then flipped into the lumen of ER, and continued the formation of oligosaccharide lipid precursor Man_9_GlcNAc_2_-PP-Dol. Afterward, the precursor transfers onto asparagine residue of the conserved Asn-X-Ser/Thr/Cys motifs (X is not proline). The newly synthesized glycoproteins are then recognized by a well-known ER quality control mechanism (ERQC) for their correct conformation (Blanco-Herrera et al., 2015). After one or multiple rounds of quality control, correct folded glycoproteins are exported from the ER to the Golgi apparatus for final mature modifications. During this ERQC mechanism, the unfolded/mis-folded glycoproteins are removed and then further processed/degraded by the ER-associated degradation pathway (ERAD) (Shin et al., 2018). The following steps of protein N-glycosylation in the Golgi apparatus are significantly different, finally giving rise to eukaryotic microalgae-specific N-glycans (Strasser, 2016). These N-glycans are classified into high mannose, paucimannose, complex, and hybrid types (Mathieu-Rivet et al., 2020).

Although the N-glycosylation pathway of proteins and mature N-glycan structures in yeasts, fungi and humans have been extensively studied. However, the information regarding the pathway of protein N-glycosylation in microalgae is still lacking. To date, the major mature N-glycans have been studied in green algae, red algae, and diatoms. For example, a complex type N-glycan bearing a core xylose residue was found in *Volvox carteri*, while the complex and oligomannosidic N-glycans were reported in *Scherffelia dubia* and *Tetraselmis striata* (Mathieu-Rivet et al., 2020). Moreover, N-glycans found in green microalga *Chlorella vulgaris* are found to be oligomannosidic N-glycans with 3-O-methyl and 3,6-di-O-methylmannose on the non-reducing terminus (Mócsai et al., 2019). The analysis of N-glycans in *Chlorella sorokiniana* demonstrated a huge heterogeneity and the presence of arabinose residues (Mócsai et al., 2020). In *Botryococcus braunii*, the synthesis of N-glycans was found to be dependent on N-acetylglucosaminyltransferase I (GnTI). Besides, it was found that N-glycans in *B. braunii* were modified by a N-acetylglucosamine (GlcNAc) residue at the non-reducing end and the (di)methylation of mannose residues (Schulze et al., 2017). The N-glycans in *C. reinhardtii* were analyzed *via* glycomic and glycoproteomic techniques which revealed that the synthesis of N-glycans is a GnTI-independent process (Mathieu-rivet et al., 2013). Moreover, oligomannosidic N-glycans (2–5 mannose residues) with 6-O-methylated mannoses and xylose residues are found to be main type in *C. reinhardtii* (Mathieu-rivet et al., 2013; Schulze et al., 2018). Later, the reinvestigation of N-glycans in *C. reinhardtii* showed that proteins carry linear Man_5_GlcNAc_2_ instead of the previous reported branched structure. It was speculated that the synthesis of linear N-glycans is owing to the lack of asparagine-linked glycosyltransferase (ALG)3, ALG6, and ALG12 activity (Vanier et al., 2017). In addition to green microalgae, O-methylated oligomannosidic N-glycan carrying xylose residues are identified from a cell wall glycoprotein of the red alga *Porphyridium* sp. (Levy-Ontman et al., 2011). High mannose type N-glycans with putative aminoethylphosphonate moieties are observed in euglenozoa *Euglena gracilis* (O’Neill et al., 2017). Moreover, it is reported that *P. tricornutum* proteins carries mainly high mannose type N-glycans (ranging from Man-5 to Man-9) in a GnTI-dependent pathway (Baïet et al., 2011). In addition to the bioinformatic analysis of genes involved N-glycosylation pathway, the reported N-glycosylation pathways in *C. reinhardtii* and *P. tricornutum* were updated and compared with the mammals in our previous study (Levy-Ontman et al., 2014; Liu et al., 2021). This comparative study revealed that N-glycosylation pathway of proteins and N-glycan structures are species-dependent. To get more reliable data, high throughput N-glycoproteomics and N-glycomics analysis have been considered to study the N-glycoproteins and N-glycans in *C. reinhardtii* (Mathieu-rivet et al., 2013), *B. braunii* (Schulze et al., 2017), and *Thalassiosira oceanica* (Behnke et al., 2021). However, the information regarding the presence of total N-glycoproteins and N-glycans in the model diatom, i.e., *P. tricornutum* still needs to be gathered.

In this study, *P. tricornutum* was used as a model organism to study the global profiling of N-glycoproteins and N-glycans using N-glycoproteomic and N-glycomic based approaches. We identified 863 N-glycopeptides in total from 639 glycoproteins in *P. tricornutum*, these proteins are involved in different cellular functions. Moreover, we identified 69 N-glycans corresponding to 59 N-glycoproteins. High mannose and hybrid type N-glycans were observed in this study. Identification of N-glycoproteins and N-glycans in *P. tricornutum* will not only deepen our understanding regarding the modification at N-glycoproteomic and N-glycomic scales but also facilitate the elucidation of the precise functions of these glycoproteins. Since it is well established that N-glycosylation affects the function and immunogenicity of recombinant pharmaceutical proteins. Therefore, this study is critically important for the expression of functional pharmaceutical N-glycoproteins in *P. tricornutum*.

## Materials and Methods

### Microalga and Growth Conditions

*Phaeodactylum tricornutum* Pt1 (Oil Crops Research Institute of Chinese Academy of Agricultural Sciences, China) cells were cultured in Erlenmeyer flasks containing f/2 medium constantly shaken at 225 rpm at 22°C under 24 h light condition (50 μmol photons m^–2^ s^–1^). Algal cells were cultured for 5 days to exponential phase in the f/2 medium. Subsequently, about 500 mg fresh algal cells were collected by centrifugation (1,500 × *g*, 10 min) for the next experiments.

### Protein Extraction

Algal cells were grinded by liquid nitrogen into cell powder and transferred into 5 ml Eppendorf tube. Subsequently, lysis buffer, including 8 M urea, 1% Triton-100, 10 mM dithiothreitol, and 1% protease inhibitor cocktail (PIC), was added to the algal powder in a ratio of 1:4. The mixture was then sonicated 3–4 times on ice using a high intensity ultrasonic processor (Scientz). After the sonication, the debris was discarded by centrifugation at 20,000 × *g*, 4°C for 10 min. The protein from supernatant was precipitated with cold 20% TCA at −20°C for 2 h. The supernatant was removed after the centrifugation at 12,000 × *g*, 4°C for 10 min. The precipitation was washed three times using the cold acetone and then dissolved in 8 M urea. The protein concentration was measured with BCA kit according to the instructions of manufacturer (ab102536, Abcam, China) (Du et al., 2021).

### Trypsin Digestion

For digestion, 5 mM dithiothreitol was used to reduce the protein solution at 56°C for 30 min and 11 mM iodoacetamide was used for the alkylation of protein at room temperature for 15 min in darkness. The protein sample was then diluted by adding 100 mM TEAB to urea concentration less than 2 M. Finally, trypsin was added for the first digestion overnight at 1:50 (trypsin-to-protein) mass ratio and for a second 4 h-digestion at 1:100 (trypsin-to-protein) mass ratio (Hockin et al., 2012).

### High-Performance Liquid Chromatography Fractionation

The tryptic peptides were fractionated into fractions by high pH reverse-phase high-performance liquid chromatography (HPLC) using Thermo BetaSil C18 column (5 μm particles, 10 mm ID, 250 mm length). Briefly, the peptides were first separated with a gradient of 8–32% acetonitrile (pH 9.0) over 60 min into 60 fractions. Then, the peptides were combined into four fractions and dried by vacuum centrifuging (Kajiura et al., 2012).

### Enrichment of Glycopeptides

The peptide mixtures were first dissolved in a loading buffer (80% acetonitrile/1% trifluoroacetic acid). The supernatant was transferred to hydrophilic interaction liquid chromatography (HILIC) microcolumn and centrifuged at 4,000 × *g* for 15 min. After three times washing with an enrichment buffer, the glycopeptides were collected from HILIC microcolumn by 10% acetonitrile and dried by vacuum freezing (Xiao et al., 2020). After drying, the glycopeptides were dissolved and digested in 50 μl 50 mM NH_4_CO_3_ buffer and 2 μl PNGase F at 37°C overnight. The digested glycopeptides were washed by C18 ZipTips and lyophilized for liquid chromatography tandem mass spectrometry (LC-MS/MS) analysis.

### N-Glycoprotein Analysis *via* Liquid Chromatography Tandem Mass Spectrometry

The tryptic peptides were dissolved in 0.1% formic acid, directly loaded onto a home-made reversed-phase analytical column (15-cm length, 75 μm i.d.). The gradient was comprised of solvent A (0.1% formic acid in 2% acetonitrile), and an increase from 9 to 25% solvent B (0.1% formic acid in 90% acetonitrile) at 0–24 min, 25 to 35% solvent B in 24–32 min, and climbing to 35 to 80% in 33–36 min then, holding at 80% for the last 4 min (36–40 min), all at a constant flow rate of 350 nl/min on an EASY-nLC 1000 UPLC system (O’Neill et al., 2017).

The peptides were subjected to nano spray ionization (NSI) source followed by MS/MS in Q Exactive™ Plus (Thermo Fisher Scientific, Waltham, MA, United States) coupled online to the ultra-performance liquid chromatography (UPLC). The electrospray voltage applied was 2.0 kV. The m/z scan range was 350–1,800 for full scan, and intact peptides were detected in the Orbitrap at a resolution of 70,000. The peptides were then selected for MS/MS using normalized collision energy (NCE) setting as 28 and the fragments were detected in the Orbitrap at a resolution of 17,500. A data-dependent procedure that alternated between one MS scan followed by 20 MS/MS scans with 15.0 s dynamic exclusion. Automatic gain control (AGC) was set at 5E4. Signal threshold was set at 5,000 ions/s, the maximum injection time was set at 200 ms. Fixed first mass was set as 100 m/z.

### N-Glycoprotein Database Search

The resulting MS/MS data were processed using MaxQuant search engine (v.1.5.2.8)^1^. The version of database is Uniprot_*Phaeodactylum*_*tricornutum*_strain_CCAP_1055 (10,465 entries). Tandem mass spectra were searched against *P. tricornutum* protein database concatenated with reverse decoy database. Trypsin/P was specified as cleavage enzyme allowing up to two missing cleavages. The mass tolerance for precursor ions was set as 20 ppm in the first search and 5 ppm in the main search, and the mass tolerance for fragment ions was set as 0.02 Da. Carbamidomethyl on Cys was specified as fixed modification and N-acetylated modification and Deamidation ^18^O (N), Deamidation (NQ) were specified as variable modifications. False discovery rate (FDR) was adjusted to <1% and minimum score for modified peptides was set at >40. Motif analysis was carried out by MoMo (v5.0.2, motif-x algorithm)^2^ (Cheng et al., 2019). And all the database protein sequences were used as background database parameter. Minimum number of occurrences was set to 20. Emulate original motif-x was ticked, and other parameters with default. Additionally, the annotations of GO and Domain were performed *via* InterProScan (v.5.14-53.0)^3^ (Blum et al., 2021), and Kyoto Encyclopedia of Genes and Genomes (KEGG) annotation were performed by using KAAS (v.2.0)^4^ and KEGG Mapper (V2.5)^5^ (Moriya et al., 2007; Kanehisa and Sato, 2020). Subcellular localization was predicted by several programs, such as SignalP 5.0^6^, TargetP 2.0^7^, ASAFind^8^, Cell-PLoc 2.0^9^, HECTAR v1.3^10^, and WoLF PSORT^11^. An enrichment analysis was carried out through the Perl module (v.1.31)^12^. The heatmap of enrichment was predicted *via* R Package pheatmap (v.2.0.3)^13^.

All differentially expressed modified protein database accession or sequences were searched against the STRING database version 10.5 for protein–protein interactions (PPI) (Szklarczyk et al., 2017). Only interactions between the proteins belonging to the searched data set were selected, thereby excluding the external candidates. STRING defines a metric called “confidence score” to define interaction confidence. We fetched all interactions that had a confidence score >0.7 (high confidence). Interaction network form STRING was visualized in R package ‘‘networkD3’’ (v.0.4)^14^.

The data will be available online^15^, the private project number is PXD022483.

### N-Glycans Analysis *via* C18-Reversed Phase Liquid Chromatography Tandem Mass Spectrometry

About 2 g fresh microalgal cells were used to protein extraction, denaturation, reduction, alkylation, and digestion with trypsin. The digested peptides were then desalted using homemade C18 SPE tips and dried in a SpeedVac. Intact N-glycopeptides were enriched using ZIC-HILIC SPE tips (Liu et al., 2017; Xue et al., 2018). The enriched intact N-glycopeptides were subsequently reductively di-ethylated with acetaldehyde and acetaldehyde-^13^C_2_ (Fang et al., 2016). The isotopic labeled intact N-glycopeptides were desalted by a C18 SPE column, dried in the SpeedVac, and dissolved with ultrapure water (18.4 MΩ cm, Millipore Simplicity System) for further C18-RPLC-MS/MS (HCD) analysis.

Mass Spectrometry spectra were acquired in the m/z range 700–2,000 with mass resolution 60 k (m/z 200). The automatic gain control (AGC) target value and maximum injection time were placed at 3 × 10^6^ and 20 ms. For MS/MS spectra, the mass resolution was set at 30 k. Fragmentation was obtained in a data-dependent acquisition (DDA) top 20 using HCD with stepped NCE (20, 30, and 31%). The AGC target value and maximum injection time were placed at 5 × 10^5^ and 250 ms. Isolation window and dynamic exclusion were set at 3.0 m/z and 20.0 s.

## Results

### Identification of N-Glycoproteins in *P. tricornutum*

**FIGURE 1:**
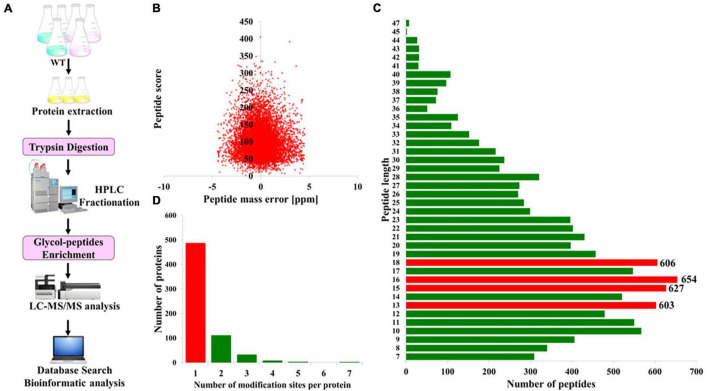
Overview of the global identification of N-glycoproteins in *Phaeodactylum tricornutum.* **(A)** General workflow of the N-glycoprotein analysis. **(B)** Mass error distribution of the identified N-glycopeptides. **(C)** Length distribution of the N-glycopeptides. **(D)** The number of N-glycosylation modification sites per protein.

To acquire a global view of N-glycosylated proteins in *P. tricornutum*, total proteins were extracted and then digested with trypsin and fractionated by HPLC. The enriched N-glycopeptides were subsequently analyzed by LC-MS/MS with MaxQuant to classify the N-glycopeptide sequence with a maximum false discovery rate of 1% (Figure 1A). In total, 863 different N-glycosylated peptides corresponding to 639 proteins were identified. The detailed information is provided in Supplementary Tables 1, 2. The mass errors of all the detected glycopeptides were distributed between −5 and +5 ppm, indicating that the mass accuracy of the MS data was fitting for the requirement of further analyses (Figure 1B). The length of the most identified N-glycopeptides was in the range of 9–23 amino acids, suggesting that the preparation of samples was standard (Figure 1C). Furthermore, it was shown that >60% of N-glycoproteins were modified at only one N-glycosylated site, few at two or multiple N-glycosylated sites (Figure 1D).

### Motif Characters of the N-Glycopeptides

**FIGURE 2:**
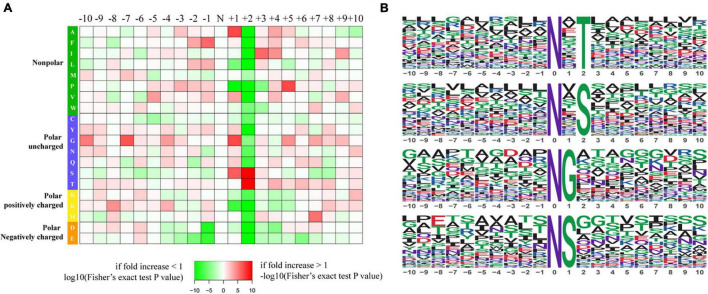
Characteristics of the N-glycosylation sites. **(A)** The motif enrichment heatmap of upstream and downstream amino acids of all identified modification sites. Red indicates that this amino acid is significantly enriched near the modification site, and green indicates that this amino acid is significantly reduced near the modification site. **(B)** The main motifs modified by N-glycans.

To further investigate the sequence models involving specific amino acids in all N-glycopeptides in *P. tricornutum*, flanking amino acid residues from position −10 to +10 around the N-glycosylated asparagine were analyzed by using a heatmap. The identified N-glycosylated peptides were analyzed in motif-X software to extract the overrepresented motifs of amino acids (Figure 2). Finally, four N-glycosylated motifs were obtained with the stringent significance. Strong bias in amino acids of specific N-glycosylated site motifs is observed. The sequence logos revealed that threonine (T) and Serine (S) are significantly overrepresented at +2 position (motif score >16), while glycine (G) and Serine (S) at +1 position (motif score >9.0) (Figures 2A,B). All the three amino acids are polar uncharged. For the identified 863 N-glycopeptides, 57.9% of the total N-glycosylation sites matched N-X-T (where X is a residue other than proline), with such sites being more abundant than those matching N-X-S (29.3%), N-G (6.8%), and N-S (6.0%). The modified site feature sequence and its detailed enrichment statistics are shown in Table 1.

**TABLE 1:** The feature sequences of modified sites.

Motif	Motif score	Foreground		Background		Fold increase
		Matches	Size	Matches	Size	
xxxxxxxxxx_N_xTxxxxxxxx	16.00	425	893	10867	168907	7.4
xxxxxxxxxx_N_xSxxxxxxxx	16.00	215	468	14090	158040	5.2
xxxxxxxxxx_N_Gxxxxxxxxx	10.12	50	253	10380	143950	2.7
xxxxxxxxxx_N_Sxxxxxxxxx	9.03	44	203	10622	133570	2.7

### Functional Annotation and Enrichment of N-Glycoproteins

**FIGURE 3:**
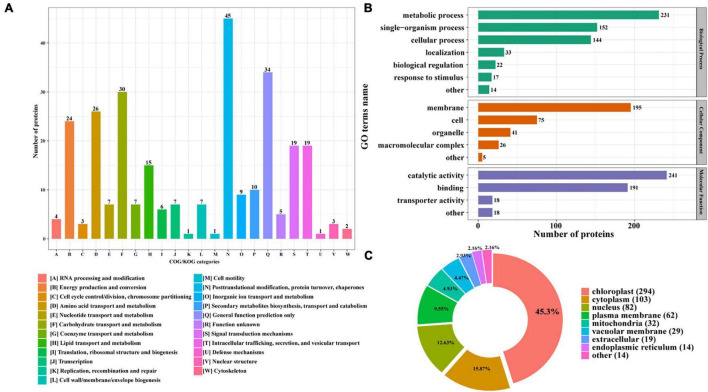
Functional distribution and subcellular localization of the N-glycoproteins. **(A)** Clusters of Orthologous Group/Eukaryote Orthologous Group (COG/KOG) functional classification chart of N-glycoproteins. **(B)** Statistical distribution chart of N-glycoproteins under each GO category (second level). **(C)** Subcellular localization chart of N-glycoproteins.

Furthermore, the identified N-glycoproteins were all annotated by Clusters of Orthologous Group/Eukaryote Orthologous Group (COG/KOG) database (Figure 3A; Zheng et al., 2020). A total of 285 N-glycosylated proteins were annotated in COG/KOG database. These identified N-glycoproteins were mainly classified into five groups. 45 N-glycoproteins (15.79%) in posttranslational modification, protein turnover, and chaperones were identified. In total, 34 (11.93%) N-glycoproteins were annotated to be general function prediction only. About 30 (10.53%) N-glycoproteins were involved in carbohydrate transport and metabolism, and 26 (9.12%) N-glycoproteins were related with amino acid transport and metabolism. Additionally, 24 (8.42%) N-glycoproteins were identified into the energy production and conversion class (Figure 3A). The gene ontology (GO) analysis was also carried out to gain an insight into more implications of these N-glycoproteins in *P. tricornutum*. All N-glycosylated proteins were annotated and classified into three categories, such as biological process, cellular component, and molecular function (Figure 3B). In the biological process category, the most prevalent GO terms were metabolic process (231 N-glycoproteins), single-organism process (152 N-glycoproteins), and cellular process (144 N-glycoproteins) (Figure 3B). In the cellular component, 195 N-glycoproteins were related with membrane. In the molecular function class, it is found that most N-glycosylated proteins are involved in the catalytic activity and binding.

The identified N-glycoproteins were classified based on subcellular localization, as shown in Figure 3C. According to the annotation, it is shown that 639 N-glycoproteins were putatively located to eight subcellular organelles, such as chloroplast, cytoplasm, nucleus, plasma membrane, mitochondria, vacuolar membrane, extracellular, and ER. Among them, most N-glycoproteins were targeted to chloroplast (294 N-glycoproteins) and cytoplasm (103 N-glycoproteins) (Figure 3C).

**FIGURE 4:**
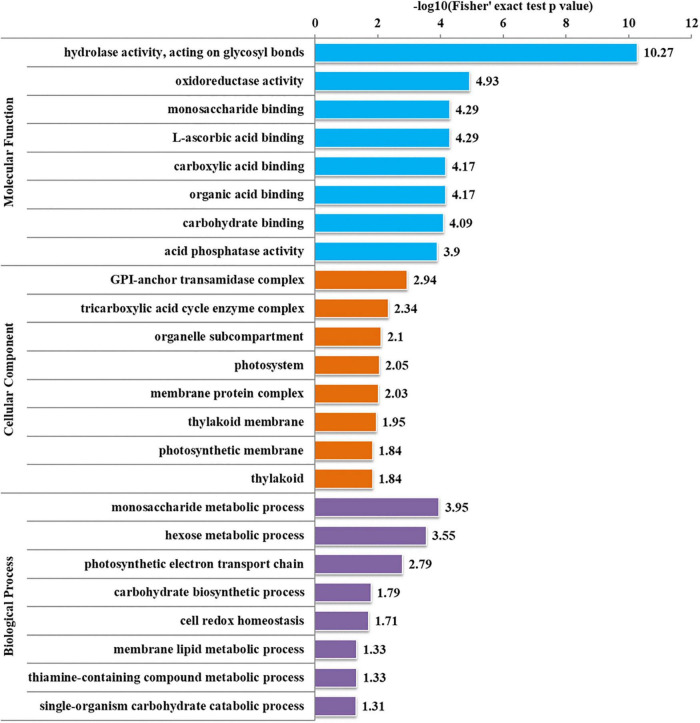
Enrichment analysis of the N-glycoproteins based on Gene Ontology (GO) annotation. Gene ontology annotation based on the three main catteries: “Biological Process,” “Cellular Component,” and “Molecular Function.” –log10 (Fisher’s exact test *p*-value): *p*-value is adjusted by Fisher. The −log10 (Fisher’ exact test *p* value) is higher, the enrichment is more significant.

**FIGURE 5:**
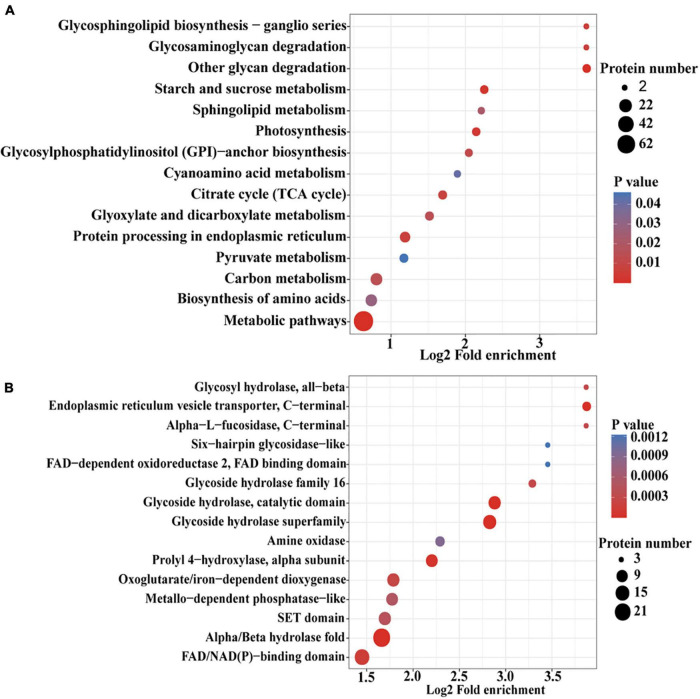
An enrichment analysis of the N-glycoproteins based on the Kyoto Encyclopedia of Genes and Genomes (KEGG) pathways and protein domains in *P. tricornutum.* **(A)** Enrichment of N-glycoproteins based on KEGG pathways. **(B)** Enrichment of N-glycoproteins based on protein domains.

To further determine the proteins more prone to be N-glycosylation, the N-glycoproteins were enriched against all proteins from *P. tricornutum* genome database. The enrichment result of N-glycoproteins based on GO annotation is shown in Figure 4. All N-glycoproteins were annotated to three categories, molecular function, cellular component, and biological process. In molecular function category, most N-glycoproteins were enriched in hydrolase activity and acting on glycosyl bonds group. In cellular component category, glycosylphosphatidylinositol (GPI)-anchor transamidase complex related proteins were more prone to be N-glycosylation. While in biological process category, the proteins involved in monosaccharide metabolic process and hexose metabolic process were thought to be easier N-glycosylated. The analysis of KEGG pathway and protein domain enrichments are shown in Figure 5. In total, 171 proteins were identified to be involved in the KEGG pathways. The most abundant KEGG pathways were carbon and nitrogen metabolic pathways with 80 N-glycoproteins, accounting for 47% of all enriched proteins (Figure 5A). Besides, 175 proteins were enriched based on protein domains (Figure 5B). It was observed that proteins containing alpha/beta hydrolase fold (25, 14%), FAD/NAD(P)-binding domain (17, 10%), and glycoside hydrolase superfamily (13, 7%) domains were preferred to be N-glycosylated.

### Interaction Networks of N-Glycoproteins

**FIGURE 6:**
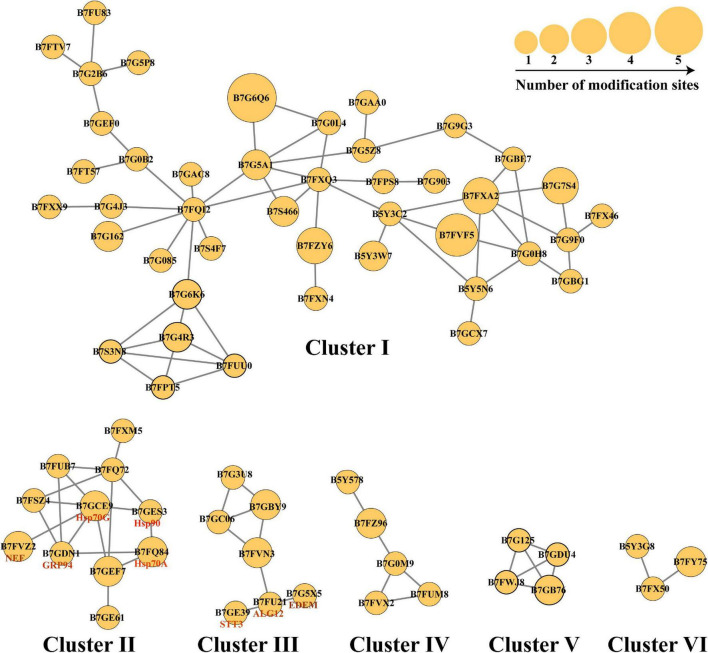
The protein–protein interaction (PPI) networks. The PPI networks were divided into six interactional clusters, from cluster I to cluster VI. Each node is a N-glycoproteins. In total, 74 N-glycoproteins were identified from six clusters. B7FXM5 is mitochondrial chaperonin. B7FUB7 is endoplasmic reticulum (ER) luminal binding protein (BiP). B7FQ72 is mitochondrial chaperonin, Cpn60/Hsp60p. B7FSZ4 is a predicted protein. B7GEF7 is Heat shock protein Hsp90. B7GE61 is Heat shock protein Hsp70. B7G3U8 is a predicted protein. B7GBY9 is glycosylphosphatidylinositol (GPI)-anchor transamidase. B7GC06 is GPI transamidase complex, GPI16/PIG-T component, involved in GPI anchor biosynthesis. B7FVN3 is mannosyltransferase. The number represents the number of modified sites.

N-linked glycosylation can significantly change the conformation of proteins and subsequently influence the interaction and function of proteins (Delgado-Vélez et al., 2021). Therefore, the analysis of N-glycoproteins interaction is important for the functional study of these N-glycoproteins. In this study, the PPI networks for all N-glycosylated proteins were established (Figure 6). A total of 74 N-glycoproteins were identified as nodes and connected as six interactional clusters (I–VI), as shown in Figure 6. The size of the node was the key parameter to evaluate the number of N-glycosylated modification sites. The biggest cluster was cluster I, containing 44 N-glycoproteins. Among them, glutamine synthase (GS, B7G6Q6) showed the largest size (five modification sites). Fumarate reductase flavoprotein (B7FVF5) showed the second-largest size (four modification sites). The cluster I contained several nodes involving in the biosynthesis of amino acids, such as glutamine synthase (GLNA, B7G6Q6), glutamine synthetase (GS, B7G5A1), and tryptophan synthase subunit beta (TrpB, B7FQI2). Glyceraldehyde-3-phosphate dehydrogenase, cytosolic class II aldolase, fructose-bisphosphate aldolase, and phosphoglycerate mutase were annotated to be involved in glycolytic pathway. Transketolase is part of the Calvin cycle. Furthermore, 11, 7, 5, 4, and 3 N-glycoproteins were observed in clusters II, III, IV, V, and VI, respectively. The cluster V harbored four N-glycoproteins, involving in starch and sucrose metabolism. They were glycoside hydrolase (ID: 56506), beta-glucosidases (ID: 26742 and ID: 50351), and exo-1,3-beta-glucosidase (ID: 49610), respectively (Figure 6).

### N-Glycoproteins Involved in the Proposed N-Glycosylation Pathway

**FIGURE 7:**
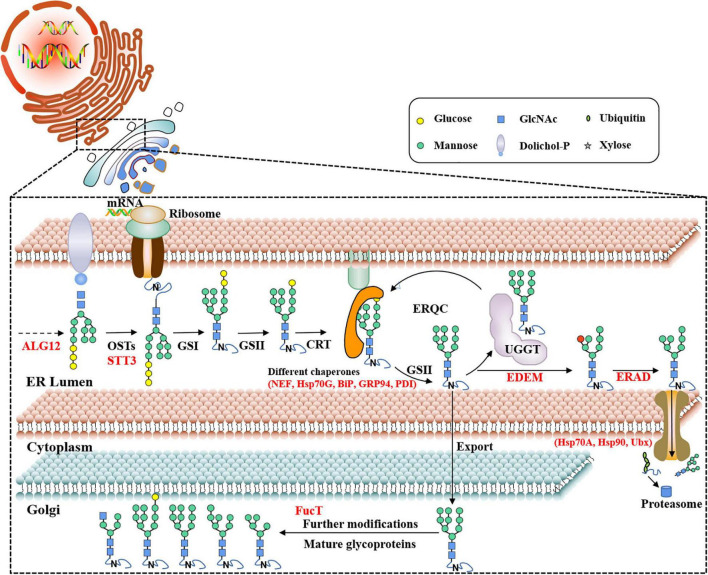
Proposed protein N-glycosylation pathway and relevant N-glycoproteins in *P. tricornutum*. ALG12, asparagine-linked glycosyltransferase 12; OST, oligosaccharyltransferase complex; STT3, staurosporine and temperature-sensitive 3; UGGT, UDP-glucose: glycoprotein glucosyltransferase; CRT, calreticulin; GS I/II, glucosidase I/II; ERQC, ER quality control; EDEM, ER degradation-enhancing α-mannosidase-like protein; ERAD, ER-associated degradation; NEF, nucleotide exchange factor; Hsp70G, Heat shock protein 70 G; BiP, binding protein; GRP94, glucose regulating protein 94; PDI, protein disulfide isomerase; Hsp70A, Heat shock protein 70 A; Hsp90, Heat shock protein 90; Ubx, ubiquitin regulatory X; FucT, core α-1,3-fucosyltransferase.

The pathway of protein N-glycosylation occurred in the ER and Golgi apparatus (Figure 7). In this study, 12 proteins involved in the N-glycosylation pathway are identified as N-glycoproteins, as shown in Table 2. Among them, ALG12, staurosporine and temperature-sensitive 3 (STT3) of oligosaccharyltransferases (OSTs) complex, binding protein (BiP), glucose regulating protein 94 (GRP94), protein disulfide isomerase (PDI), ER degradation-enhancing α-mannosidase-like protein (EDEM), Heat shock protein 90 (Hsp90), and core α-1,3-fucosyltransferase (FucT) N-glycoproteins contained one N-glycosylated modification site. While nucleotide exchange factor (NEF), Heat shock protein 70 G (HSP70G), Heat shock protein 70 A (HSP70A), and ubiquitin regulatory X (Ubx) N-glycoproteins harbored two N-glycosylated modification sites in *P. tricornutum*. ALG12, STT3 of OSTs complex, NEF, HSP70G, BiP, GRP94, and PDI are proposed to be involved in the ER pathway of protein N-glycosylation. However, FucT being putatively located to the Golgi apparatus, relating with the Golgi pathway of protein N-glycosylation. EDEM, Hsp90, HSP70A, and Ubx belonged to the ERAD pathway. Based on the prediction of PPI networks, it is found that eight identified N-glycoproteins involved in protein N-glycosylation pathway are predicted to two interactional complexes (Figure 6). In the cluster II, NEF and Hsp70G, Hsp70G and Hsp90, Hsp90 and Hsp70A proteins might interact, respectively. GRP94 protein is proposed to interact with Hsp70G and Hsp70A proteins, but not with Hsp90. In the cluster III, STT3 and ALG12 might interact each other, ALG12 and EDEM proteins might interact each other.

**TABLE 2:** The detailed information of N-glycoproteins during the protein N-glycosylation pathway.

Protein name	Protein ID	Amino acid position	Localization probability	Modified sequence
ALG12	44425	527	1	VSNSSTYTHMLSESK
OSTs (STT3)	55198	826	0.96	MWNLINSNAVEELK
NEF	44879	317	1	NTGEFAPWALEELTLGNESSIAR
NEF	44879	56	1	EWTLLRENDTVAAGMHVR
Hsp70G	55122	126	1	HYPVRPVYNETR
Hsp70G	55122	435	1	HINSDESMALGAAFAGANISTAFR
BiP	54246	567	1	NGLESYLYNLK
GRP94	16786	41	1	ELISNASDALDKFR
PDI	2808	169	1	GVLANVSK
EDEM	52346	116	1	VDRNVSVFETNIR
Hsp70A	54019	356	1	VQSMLSEFFNGKEPCK
Hsp70A	54019	63	0.99	SQAAMNAHNTVFDAK
Hsp90	55230	95	0.63	VDLVNNLGTIAK
Ubx	34061	59	1	GGGSGLAVQPNMDEGPDRD
Ubx	34061	43	1	AVFGLAENATAEDSGQSR
FucT	46109	554	1	EFGLGWNHTAQTIQPTHLPR

### N-Glycans Profiling

**FIGURE 8:**
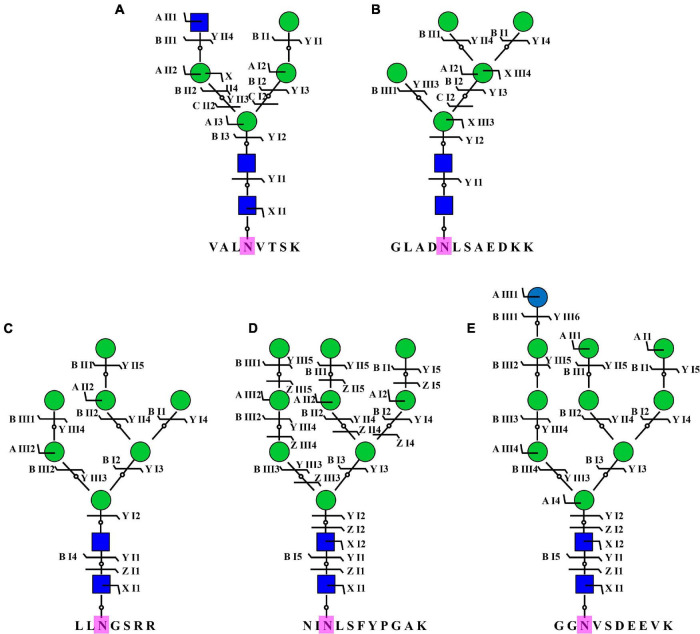
Representative N-glycans identified in *P. tricornutum.* Five representative N-glycans were from five different N-glycoproteins, including B7FVG6 **(A)**, B7FUB7 **(B)**, B7FUL9 **(C)**, B7FUW2 **(D)**, and B7FTM0 **(E)**. Blue square is N-acetylglucosamine. Green circle is mannose. Blue circle is glucose. Purple N is asparagine amino acid for the N-glycosylation.

To acquire a global view of N-glycans in *P. tricornutum*, total proteins were extracted and digested by trypsin, and the digested peptides were desalted using homemade C18 SPE tips. The enriched N-glycopeptides *via* ZIC-HILIC SPE tips were subsequently isotopic labeled and analyzed by using C18-RPLC-MS/MS (HCD). Totally, 69 N-glycans corresponding to 59 N-glycoproteins were identified from this study. The detailed data are shown in Supplementary Table 3. All 69 N-glycans were divided into two classes, high mannose, and hybrid type N-glycans. One hybrid type N-glycan with a GlcNAc was identified as GlcNAc_1_Man_4_GlcNAc_2_ (Figure 8A). Sixty-eight high mannose type N-glycans were identified, such as three Man_5_GlcNAc_2_ (4.35%, Man, mannose; GlcNAc, N-acetylglucosamine) (Figure 8B), three Man_7_GlcNAc_2_ (4.35%) (Figure 8C), 55 Man_9_GlcNAc_2_ (79.71%) (Figure 8D), and seven Glc_1_Man_9_GlcNAc_2_ (10.14%, Glc, glucose) (Figure 8E).

## Discussion

We used an integrated N-glycoproteomic and N-glycomic approach to unravel the protein N-glycosylation pathway of *P. tricornutum* and shed light on N-glycans structure and N-glycoproteins. This approach has been used in limited studies so far. So far, the N-glycoproteomic analysis was performed in three green algae *C. reinhardtii* (Mathieu-rivet et al., 2013), *B. braunii* (Schulze et al., 2017), and diatom *T. oceanica* (Behnke et al., 2021). Compared with the identified 135 N-glycopeptides in *C. reinhardtii*, total 517 unique N-glycosylated peptides in three strains of *B. braunii* and 118 N-linked glycosylated peptides in *T. oceanica*, 863 different N-glycopeptides corresponding to 639 proteins were identified in this study in of *P. tricornutum.* The large difference on the number of the identified N-glycopeptides might be related with different methods. In *C. reinhardtii* and three *B. braunii* strains, the enrichments of N-glycopeptides were performed by using agarose-bound concanavalin A lectin, and the analysis methods of N-glycopeptides were PNGase F/^18^O-method and in-source collision induced dissociation (IS-CID) methods (Mathieu-rivet et al., 2013) and LC-MS (Schulze et al., 2017), respectively. Furthermore, solid–phase extraction, hydrophilic-lipophilic balance (HLB) cartridge enrichment, LC-MS/MS analysis were carried out in the N-glycopeptide analysis of *T. oceanica* (Behnke et al., 2021). These enrichment methods are N-glycan-specific, especially the lectin enrichment, while HILIC microcolumn was considered in the current to get more enriched N-glycopeptides. But still, few known N-glycoproteins remained identified, such as tRNA synthetase (ID: 43097), the putative mismatch repair protein MutS (ID: 47730), and symbiont-specific ubiquitin fusion degradation protein 1 (ID: 49319) (Peschke et al., 2013), indicating that the analysis method of N-glycoproteins still needs to be improved.

In this study, we found that the canonical N-X-T motifs accounted for 57.9% of all motifs while in diatom *T. oceanica*, the identified peptides had 81% N-X-T motifs (Behnke et al., 2021), indicating that N-X-T motif is the main N-glycosylation site in diatom, and its percentage varies species to species. The presence of main N-glycoproteins in chloroplast and cytoplasm indicates the regulatory role of N-glycosylation in chloroplast and cytoplasm. It is already known that N-glycosylation modification could directly affect the subcellular localization of proteins, especially for proteins passing through the ER-Golgi network (Friso and Van Wijk, 2015). Therefore, it is proposed that the N-glycosylation plays an important role in the subcellular localization of these 639 N-glycoproteins. The subcellular localization of some identified N-glycoproteins had already been verified by experimental data in *P. tricornutum*. The first one was aminoacyl-tRNA synthetase (Glx, ID: 48863). Consistent with the prediction of subcellular localization in this study, N-glycosylated Glx was located to the plastid stroma of *P. tricornutum* (Peschke et al., 2013). It is certified that bulky glycoproteins could be transported into the complex plastid of *P. tricornutum*, and speculated that these proteins are N-glycosylated on the outermost plastid membrane and thereafter transported into the plastid (Peschke et al., 2013). Postsynaptic density protein 95, discs large, zonula occludens 1 (PDZ) domain containing glycoprotein-2 (PDZ-2, ID: 47562) was predicted to localize in plasma membrane in this study. This prediction is consistent with our previous experimental result (Liu et al., 2016). On the contrary, some predicted localizations of glycoproteins are not consistent with the experimental results. For example, the prediction showed that Omp85 is located to the vacuole, while our previous experimental result showed the third outermost membrane localization in *P. tricornutum* (Liu et al., 2016). This discrepancy indicated that the results from bioinformatic prediction need to be verified by the experiments.

Furthermore, N-glycoproteins were annotated and enriched *via* different database. The interaction networks of N-glycoproteins were predicted. These annotations, enrichments, and prediction of PPIs provide advantages for the researchers to study the functions of these N-glycoproteins in different biological processes and the effects of N-glycosylation on these N-glycoproteins (Wang et al., 2013). Here, taking glutamine synthase as an example, GS harbored five N-glycosylated sites in *P. tricornutum*. It is already known that GS was one of the most important enzymes for the nitrogen assimilation and absorption (Liu et al., 2019). GS catalyzed the assimilation of ammonium into glutamate and produced glutamine in the cytoplasm, mitochondria, and chloroplast (Konishi et al., 2016; Smith et al., 2019). The function of GS isoenzymes on the regulation of nitrogen metabolism was widely studied in different organisms, such as in microalgae *C. reinhardtii* (Cabello et al., 2020) and macroalga *G. lemaneiformis* (Liu et al., 2019). However, the N-glycosylation of GS in *P. tricornutum* is reported for the first time in this study. However, the effects of N-glycosylation on the biological functions of GS still remain unknown.

In addition to the proposed protein N-glycosylation pathway in green algae, *C. vulgaris* and *C. reinhardtii*, the pathway in *P. tricornutum* was first described *via* bioinformatic analysis (Baïet et al., 2011), showing that it was a GnTI-dependent process. Subsequently, the pathway was updated and was compared with *C. reinhardtii* and mammals. The data revealed a species specific nature of N-glycosylation pathway (Liu et al., 2021). Moreover, the genes involved in protein N-glycosylation pathway were also identified in *T. oceanica* (Behnke et al., 2021) and in other 17 microalgae (Liu et al., 2021) *via* bioinformatic methods. Although the protein N-glycosylation pathways in these microalgae are proposed, they need to be elucidated by experiments in the future. In this study, 12 N-glycoproteins involved in the protein N-glycosylation pathway were identified, such as ALG12, STT3, NEF, Hsp70G, BiP, GRP94, PDI, EDEM, Hsp70A, Hsp90, and Ubx in ER, and FucT in Golgi apparatus. It was indicated that the N-glycosylation modification of these proteins might be important for their functions during the protein N-glycosylation pathway. Two interaction networks of eight N-glycoproteins were predicted, suggesting that these N-glycoproteins might form two complexes and regulate the ERQC and ERAD in *P. tricornutum*. These results provide valuable data for studying function of genes (e.g., ALG12, STT3, and NEF) and the pathway of protein N-glycosylation. Until now, the functions of some genes involved in the protein N-glycosylation pathway have been reported. For examples, the heterologous expression of *P. tricornutum* GnTI restored the complex type N-glycans maturation in the Chinese hamster ovary (CHO) mutant, indicating its importance on the synthesis of complex type N-glycans (Baïet et al., 2011). Subsequently, it was observed that the GDP-fucose transporter and FucT1 were involved in the fucose modification of proteins in *P. tricornutum* (Zhang et al., 2019). In addition to *P. tricornutum*, the functions of genes, such as xylosyltransferase A (XylTA), xylosyltransferase B (XylTB), fucosyltransferase (FucT), and mannosidase 1A (Man1A) were studied during the protein N-glycosylation pathway of *C. reinhardtii* (Mathieu-Rivet et al., 2020). In *Porphyridium* sp., α-1,3-Glycosidase II was proposed to act in the ER (Levy-Ontman et al., 2015). The elucidations of genes function were important for revealing the protein N-glycosylation pathway in microalgae, especially for microalgae as cell factories to produce the humanized biopharmaceutical glycoproteins.

For the N-glycan structures, it was first reported that *P. tricornutum* proteins contained mainly high mannose type N-glycans, Man_5_GlcNAc_2_ to Man_9_GlcNAc_2_ (Baïet et al., 2011). The structure of the lipid linked oligosaccharide in *P. tricornutum* was Glc_2_Man_9_GlcNAc_2_ (Lucas et al., 2018). These high mannose type N-glycans were further confirmed by IMS-MS and ESI-MS*^n^* fragmentation patterns data (Dumontier et al., 2021). Subsequently, it was demonstrated that the proteins in *P. tricornutum* were N-glycosylated by single isomers of Man-5, Man-6, and Man-9 mannosides, and three isomers of Man-7 and Man-8 mannosides (Dumontier et al., 2021). Owing to the synthesis of canonical Man-5 and a GnTI-dependent pathway, it was speculated that *P. tricornutum* could initiate the processing of oligomannosides into complex type N-glycans (Baïet et al., 2011; Zhang et al., 2019). In this study, Man-5, Man-7, Man-9, and Man-10 mannose type N-glycans and a hybrid N-glycan with a terminal GlcNAc residue were identified. However, different isomers were not found from these N-glycans. The more importance was that this study updated the N-glycans database of *P. tricornutum*, owing to the new reported N-glycans, such as Glc_1_Man_9_GlcNAc_2_ and GlcNAc_1_Man_4_GlcNAc_2_. Compared with the N-glycans reported in the previous studies, methylated mannose, linear Man_5_GlcNAc_2_, and complex N-glycans with xylose and/or fucose residues were not observed in this study because of two reasons. One reason might be owing to the loss caused by experimental operations. The other reason might be that PNGase F could not cleave complex N-glycans with fucose and/or xylose modified core structure (Song et al., 2013). Although PNGase A could cleave all types of asparagine binding N-glycans, the efficiency was very low (Song et al., 2013). Therefore, an appropriate identification method for N-glycans is very important.

Asparagine-linked glycosyltransferase 12, a N-glycoprotein, was the first enzyme in protein N-glycosylation pathway to be identified. ALG12 gene was predicted from the genome of most organisms, such as red microalga *Galdieria sulphuraria*, diatom *P. tricornutum*, green microalga *Ostreococcus lucimarinus*, and *Chlorella*, however, ALG12 was not annotated in the genomes of red microalgae *Cyanidischyzon merolae* and green microalgae *C. reinhardtii* (Levy-Ontman et al., 2014). It is reported that ALG12 is important for the formation of N-glycan structures, as the lack of ALG12 in *C. reinhardtii* the branched N-glycan is substituted by the linear oligomannosidic N-glycan (Mathieu-rivet et al., 2013). The mutation of ALG12 affected the transfer of mannose to N-glycan structure (Dempski and Imperiali, 2002). Additionally, it was indicated that ALG12 affected the N-glycan structure, but not the cell growth and viability (Kajiura et al., 2010). Therefore, it was speculated that the N-glycosylation of ALG12 might play an important role in the N-glycan structures in *P. tricornutum*. In conclusion, the exploration of the *P. tricornutum* N-glycoproteins, N-glycans, and the proposed N-glycosylation pathway as done in this study represents an important first step toward the design of genetically engineered driven remodeling of the alga to produce functional humanized biopharmaceutical N-glycoproteins for the clinical therapeutics.

## Data Availability Statement

The datasets presented in this study can be found in online repositories. The names of the repository/repositories and accession number(s) can be found in the article/Supplementary Material.

## Author Contributions

XL designed the experiments and supervised the project. XX, HoD, JC, MA, WW, WC, PL, HuD, and XL performed the experiments and the revised manuscript. XL and MA wrote the draft manuscript. All authors discussed the results and implications and commented on the manuscript at all stages.

## Conflict of Interest

The authors declare that the research was conducted in the absence of any commercial or financial relationships that could be construed as a potential conflict of interest.

## Publisher’s Note

All claims expressed in this article are solely those of the authors and do not necessarily represent those of their affiliated organizations, or those of the publisher, the editors and the reviewers. Any product that may be evaluated in this article, or claim that may be made by its manufacturer, is not guaranteed or endorsed by the publisher.

## Footnotes

1
http://www.maxquant.org/

2
http://meme-suite.org/tools/momo

3
http://www.ebi.ac.uk/interpro/

4
http://www.genome.jp/kaas-bin/kaas_main

5
http://www.kegg.jp/kegg/mapper.html

6
http://www.cbs.dtu.dk/services/SignalP/

7
http://www.cbs.dtu.dk/services/TargetP/

8
https://rocaplab.ocean.washington.edu/tools/asafind/

9
http://www.csbio.sjtu.edu.cn/bioinf/Cell-PLoc-2/

10
https://webtools.sb-roscoff.fr/?tool_id=toolsheddev.sb-roscoff.fr%2Frepos%2Flgueguen%2Fhectar_3_0%2Fabims_hectar%2F1.2&version=1.2&__identifer=vx6suh9fhag

11
https://wolfpsort.hgc.jp/

12
https://metacpan.org/pod/Text:NSP:Measures:2D:Fisher

13
https://cran.r-project.org/web/packages/cluster/

14
https://cran.r-project.org/web/packages/networkD3/

15
http://beta.uniprot.org/
